# Preliminary Empirical Validation of the “Basic Needs Satisfaction in Sport Scale” With a Sample of Spanish Athletes

**DOI:** 10.3389/fpsyg.2018.01057

**Published:** 2018-06-27

**Authors:** Cristina De Francisco, Francisco J. Parra, Constantino Arce, M. D. Pilar Vílchez

**Affiliations:** ^1^Faculty of Social Sciences and Communication, Catholic University of Murcia, Murcia, Spain; ^2^Department of Social Psychology, Basic and Methodology, University of Santiago de Compostela, Santiago de Compostela, Spain

**Keywords:** basic psychological needs, sport, Spanish, validity, reliability

## Abstract

The theory of self-determination establishes the existence of three basic psychological needs (autonomy, competence, and relationship). If these needs are satisfied, optimal personal well-being will be achieved. The *Basic Needs Satisfactions in Sport Scale* (BNSSS) is a measurement developed to evaluate these needs within the sporting context. The BNSSS measures the satisfaction of the three basic psychological needs through 20 items distributed in five dimensions: autonomy-choice, autonomy-volition, autonomy-perceived locus of internal causality, competence, and relatedness. The purpose of this study is to validate a Spanish version of the BNSSS. The sample were 441 team athletes with a mean age of 17.46 (*SD* = 3.59), which 46.5% were men and the remaining percentage (53.5%) were women. After a standardised data collection, confirmatory factor analysis and invariance analyses were performed, as well as composite reliability. The obtained version showed a good overall fit of the model and values of composite reliability higher to 0.70. Therefore, a useful tool for assessing basic psychological needs in team sports was obtained.

## Introduction

In recent years, there has been a great deal of research regarding self-determination theory in a sporting context (Podlog and Eklund, 2007; Ryan and Deci, 2007; Wilson et al., 2008; Ryan et al., 2009; Pelletier et al., 2013; Wang et al., 2016). The theory also encompasses several others explaining what motivates athletes and their behaviour, one example being the theory of basic psychological needs (Ryan and Deci, 2000). Ryan (1995) asserted we have three basic psychological needs – autonomy, competence, and relatedness – all of which must be satisfied to achieve an optimal level of well-being. According to Chen et al. (2015), if these needs are not met, then it could cause general personal distress and even result in certain psychological problems.

The need for *autonomy* refers to a subject’s need to feel as if they are the cause of their behaviour or carry out actions according to their own will (deCharms, 1968). The need for *competence* concerns an individual’s capacity to feel effective in their behaviour or to complete tasks with different levels of difficulty (Deci, 1971). Finally, the need for *relatedness* refers to the feeling or sensation of being connected, supported, or loved by others (Ryan, 1995). According to Ryan and Deci (2000), these needs apply to all individuals, regardless of age, sex, or culture.

These three needs have a direct influence on a sportsperson’s motivation as the perception of satisfying them generates a state of self-determination or greater degree of intrinsic motivation. Different studies have concluded that the fulfilment of these needs is a predictor of intrinsic motivation and this satisfaction is positively associated with well-being, both in a general (Reis et al., 2000; Sheldon and Niemiec, 2006; Ryan et al., 2010; Sheldon and Hilpert, 2012; Chen et al., 2015), and in a sporting context (Reinboth et al., 2004; Wilson and Rodgers, 2004; Reinboth and Duda, 2006; Ng et al., 2011). Contrastingly, the frustration of these needs impacts negatively on people’s psychological health and well-being, causing low levels of motivation or even demotivation (Vansteenkiste and Ryan, 2013). Furthermore, satisfaction of the basic psychological needs has a negative correlation with constructs such as depression (Wei et al., 2005), anxiety (Deci et al., 2001), or burnout syndrome (Perreault et al., 2007; Hodge et al., 2008).

Consequently, Gagné (2003) outlined the need to study the factors affecting motivation and measure basic psychological needs satisfaction. Hence, in 2003, he developed the Basic Psychological Needs at Work Scale (BPNWS), based on previous research published by Ilardi et al. (1993) and Deci et al. (2001), which is currently one of the most popular scales for measuring basic psychological needs satisfaction in the workplace.

With regards to physical activity, Vlachopoulos and Michailidou (2006) created the Basic Psychological Needs in Exercise Scale, comprising 12 items, four for each dimension (competence, autonomy, and relatedness), that solicited responses according to a Likert scale ranging from 1 (strong disagreement) to 5 (strong agreement). The tool’s internal consistency generated Cronbach’s alpha coefficients of 0.81 for competence, 0.84 for autonomy, and 0.92 for relatedness. The model also presented a good fit following confirmatory factor analyses with a value of 0.97 for both the non-normed fit index (NNFI) and the comparative fit index (CFI). The root mean square error of approximation (RMSEA) was 0.05.

In the same year, Wilson et al. (2006) developed the Psychological Need Satisfaction in Exercise Scale, featuring 18 items, six for each dimension (competence, autonomy, and relatedness), based around a 6-point Likert response scale (1 = strong disagreement, 6 = total agreement). Their tool also demonstrated high levels of internal consistency: 0.91 for competence and autonomy, 0.90 for relatedness. Regarding the model’s fit, both the CFI and the incremental fit index (IFI) were 0.94, while the RMSEA reached a value of 0.09.

Both the Basic Psychological Needs in Exercise Scale and the Psychological Need Satisfaction in Exercise Scale focused on the health benefits of practising physical activity rather than sporting performance. Using samples comprising subjects who practised physical exercise as well as athletes, Gillet et al. (2008) developed the *Échelle de Satisfaction des Besoins Psychologiques*, consisting of 15 items, five for each dimension (competence, autonomy and relatedness) with a 7-point Likert type response scale, ranging from “not at all true” (1) to “very true” (7). It produced the following values for the psychometric properties: Cronbach’s alpha of 0.71 for competence, 0.82 for autonomy, and 0.81 for relatedness; while the CFI and IFI were both 0.95, the NNFI 0.93 and the RMSEA 0.06.

However, this scale did not evaluate basic psychological needs satisfaction in high-performance sports and so Ng et al. (2011) presented a tool specifically for use within a competitive sporting context, which they applied to a sample of affiliated athletes and called the Basic Needs Satisfaction in Sport Scale (BNSSS). The BNSSS contains 20 items; 10 cover autonomy and there are five each for competence and relatedness. The autonomy dimension was given more weight when Ng et al. (2011) presented their version, taking into account experts items revision, they decided to divide it into three subscales: four items for autonomy-choice (decision making opportunities), three items for autonomy-volition (unpressurised involvement), and three items for autonomy-internal perceived locus of causality (ability to regulate behaviour). The tool’s internal consistency produced Cronbach’s alpha coefficients of 0.77 for competence, 0.82 for autonomy-choice, 0.61 for autonomy-volition, 0.76 for autonomy-internal perceived locus of causality (IPLOC), and 0.87 for relatedness. The model fit indicators also revealed good results: NNFI = 0.96; CFI = 0.97; RMSEA = 0.06; and the standardised root mean square residual (SRMR) = 0.07.

The present study was designed to produce an instrument available for use in Spain that could measure basic psychological needs satisfaction in athletes. The aim was to complete a Spanish validation of the BNSSS scale in team sports. This will be achieved by taking on some specific objectives: a psychometric validation of a translation/adaptation of the original instrument, an invariance analysis by sex and age (two of the most significant sociodemographic variables) and a composite reliability analysis.

## Materials and Methods

### Participants

The intentional, non-probabilistic sample comprised 441 participants involved in different types of team sport, of which 46.5% were men and 53.5% women. The team sport with the highest participation percentage was football (40.6%), followed by basketball (16.6%) and indoor 5-a-side football (14.5%). Study participants ranged from 14 to 30 years old (*M* = 17.46; *SD* = 3.59); 62.8% of the athletes were minors and 37.2% were adults. The majority, 82.8%, were amateur athletes while the rest were elite competitors. The mean number of weekly training sessions was 3.01 (*SD* = 0.92), with an average duration of 99.54 min/session (*SD* = 24.45).

### Instrument

The Spanish version of the BNSSS was used instrument after subjecting the original scale (Ng et al., 2011) to a double translation process. Following the recommendations of Muñiz and Hambleton (2000), one translator (Spanish native), knowledgeable of the English-speaking culture, realised a translation of the items taking into a conceptual equivalent of a phrase, not a word-for-word translation. It was the forward translation. After, an independent translator, whose mother tongue was English and who had no knowledge of the questionnaire translated the instrument back to English (back translation). As in the initial translation, emphasis in the back-translation was on conceptual and cultural equivalence and not linguistic equivalence. Discrepancies were discussed by an expert panel formed by five sport psychologists.

The BNSSS incorporates 20 items: five are used to measure competence, another five items are for the relatedness dimension and 10 items assess the autonomy dimension. The latter dimension is subsequently divided into four items for autonomy-choice, while autonomy-volition and autonomy-IPLOC are both allocated three items. The scale is a Likert-type questionnaire with responses ranging from 1 “Not true at all” to 7 “Very true”. The highest numerical value indicates the highest level of satisfaction, with the exception of the fifth item (“In sport, I feel that I am being forced to do things that I don’t want to”, autonomy-volition) which was devised inversely (the highest numerical value indicates the lowest degree of satisfaction).

Also the Spanish version of the “Psychological Needs Thwarting Scale” (PNTS; Bartholomew et al., 2011) was used to assess the frustration of three basic psychological needs within the sport field (competence, autonomy, and relatedness). It was validated to the Spanish context by Sicilia et al. (2013). The PNTS is formed by 12 items, 4 items for each need. A 7-point Likert scale is used from 1 “totally disagree” to 7 “totally agree” for the responses. All items were enunciated so that to greater numerical answer, greater degree of thwarting.

In addition, the booklet also featured questions about sociodemographic factors in terms of sex, age, and training history (type of sport, years of training, duration of training sessions, number of sessions per week, and competitive level).

### Procedure

First of all, an authorization request was submitted to the university’s ethics committee, which duly approved the research procedure with written informed consent from all subjects. All subjects gave written informed consent in accordance with the Declaration of Helsinki. Then the research team contacted the participants and/or sports club administrators to arrange an appointment and to implement the booklet. One researcher visited each team at their training sites and handed out the questionnaire to the group 15 min before their normal training session. Before conducting the questionnaires, participants (or their legal tutor/s if they were minors) signed an informed consent form and then filled out the booklet.

### Data Analysis

After pruning the database to check for any out-of-range responses or atypical cases, the initial sample of 448 athletes was reduced to 441. The software programme IBM SPSS Statistics 21 was then used to calculate the descriptive statistics. Given preliminary studies into the factor structure were already carried out in the original version (Ng et al., 2011), in this paper directly confirmatory factor analysis was performed using the programme EQS 6.3. Polychoric correlation matrix was computed and the generalised weighted least squares method used to estimate the parameters of the model. The ratio between χ^2^ and its degrees of freedom (*df*) was calculated to evaluate the measurement models’ fit. Also the RMSEA which is indicative of good fit for values of less than 0.05. The NNFI and CFI were also determined for which indices of greater than 0.90 and 0.95, respectively, are recommended to obtain a good model data fit (Levy and Varela, 2006). In order to provide more evidence on the validity of the questionnaire, bivariate correlations were calculated between the BNSSS and the PNTS dimensions. In the same sense, the average variance extracted (AVE) was also computed for each factor, following the procedure indicated by Fornell and Larcker (1981). Values greater than 0.50 can be taken as evidence of convergent validity of the scale.

An invariance analysis also was performed based on three additional models to test for equality of model parameters between the groups of men and women, and minors and adults. Model 1 (configuration model) is a basic model with unrestricted parameter estimation in the various groups; Model 2 specifies the equality or invariance of the factor loadings between groups; Model 3 considers the equality between groups of factor loadings, correlations between factors and factor variances. Invariance is typically assessed by using the χ^2^ test to calculate the differences between Model 1 and Models 2 and 3. However, in the present study, we have used Cheung and Rensvold’s (2002) criterion, who advocated evaluating the difference in the CFI values instead of the χ^2^ test. Differences between models of greater than 0.01 were considered indicators of non-invariance.

For reliability analysis, the composite reliability index (Fornell and Larcker, 1981) was calculated to avoid the bias introduced with Cronbach’s alpha, which does not take into account the influence of multidimensionality (Dunn et al., 2014). Prieto and Delgado (2010) established that in order for values for this statistic to be accepted, then they must be <0.7 in descriptive cases or <0.9 in selective tests.

## Results

### Descriptive Statistics

**Table 1** shows the descriptive statistics for each item. The means lay between 4.84 (*SD* = 1.55; item 9, autonomy-choice) and 6.63 (*SD* = 0.80; item 8, autonomy-volition). The standard deviations were found to range between 0.80 (item 8, autonomy-volition) and 1.66 (item 5, autonomy-volition). With regards to the data’s distribution, all items presented negative skewness, with items 8 (autonomy-volition) and 19 (relatedness) returning the highest values (-2.80 and -2.05, respectively). Finally, the kurtosis indices were primarily positive, item 8 scored the highest value (8.78, autonomy-volition), followed by items 19 (5.07, relatedness) and 1 (3.63, relatedness).

**Table 1:** Descriptive statistics.

Items	Dimensions	Mean	Standard deviation	Skewness	Kurtosis
(1) In sport, I have a close relationship with other people.	RL	6.33	0.99	-1.79	3.63
(En el deporte, tengo una relación cercana con otra gente)					
(2) In sport, I feel I am pursuing goals that are my own.	AUT_IPLOC_	5.53	1.46	-1.13	0.85
(En el deporte, siento que las metas que persigo son las mías propias)					
(3) I feel I participate in my sport willingly.	AUT_V L_	6.17	1.03	-1.37	1.59
(Tengo la sensación de jugar al deporte de buena gana)					
(4) In sport, I get opportunities to make choices.	AUT_CH_	5.24	1.49	-0.79	0.14
(En el deporte, tengo oportunidades para elegir)					
(5) In sport, I feel that I am being forced to do things that I don’t want to do.	AUT_V L_	5.74	1.66	-1.28	0.58
(En el deporte, me siento obligado/a a hacer cosas que no quiero hacer)					
(6) I can overcome challenges in my sport.	CM	5.71	1.23	-1.25	1.86
(Soy capaz de superar desafíos en el deporte)					
(7) I show concern for others in my sport.	RL	5.78	1.44	-1.54	2.13
(Muestro preocupación por otras personas en el deporte)					
(8) I choose to participate in my sport according to my own free will.	AUT_V L_	6.63	0.80	-2.80	8.78
(Decido jugar al deporte por voluntad propia)					
(9) In my sport, I have a say in how things are done.	AUT_CH_	4.84	1.55	-0.61	-0.09
(En el deporte, mi opinión cuenta a la hora de decidir cómo se hacen las cosas)					
(10) There are people in my sport who care about me.	RL	6.15	1.08	-1.44	2.04
(En el deporte, tengo compañeros/as que se preocupan por mí)					
(11) I am skilled at sport.	CM	5.88	1.10	-0.89	0.61
(Tengo aptitudes para jugar al deporte)					
(12) I feel I am good at sport.	CM	5.49	1.24	-0.69	0.01
(Creo que soy bueno/a en el deporte)					
(13) In sport, I can take part in the decision making process.	AUT_CH_	4.95	1.54	-0.62	-0.12
(En el deporte, puedo formar parte del proceso de toma de decisiones)					
(14) I get opportunities to feel that I am good at sport.	CM	5.56	1.21	-0.69	-0.10
(Tengo oportunidades para sentir que soy bueno/a en el deporte)					
(15) In sport, I really have a sense of wanting to be there.	AUT_IPLOC_	5.92	1.21	-0.98	0.32
(En el deporte, tengo la sensación de querer estar donde estoy)					
(16) In sport, I feel I am doing what I want to be doing.	AUT_IPLOC_	6.19	1.08	-1.41	1.59
(En el deporte, tengo la sensación de estar haciendo lo que quiero hacer)					
(17) I have the ability to perform well in sport.	CM	5.77	1.10	-0.77	0.24
(Tengo capacidades para obtener buenos resultados en el deporte)					
(18) In sport, there are people who I can trust.	RL	6.30	0.96	-1.57	2.33
(En el deporte, hay gente en la que puedo confiar)					
(19) I have close relationships with people in sport.	RL	6.45	0.91	-2.05	5.07
(En el deporte, tengo buenas relaciones con mis compañeros/as)					
(20) In sport, I get opportunities to make decisions.	AUT_CH_	5.21	1.56	-0.74	-0.06
(En el deporte, tengo la oportunidad de tomar decisiones)					

*RL, relatedness; AUT_*IPLOC*_, autonomy-internal perceived locus of causality; AUT_*VL*_, autonomy-volition; CM, competence; AUT_*CH*_, autonomy-choice.*

### Confirmatory Factor Analysis (CFA) and Other Evidences of Validity

A confirmatory factor analysis (CFA) was run based on the factor structure defined by Ng et al. (2011), in which the BNSSS items were grouped into five dimensions: autonomy-choice (4 items), autonomy-volition (3 items), autonomy-IPLOC (3 items), competence, and relatedness (5 items each).

The standardised residuals varied from -0.35 (ratio between items 16 and 12 – autonomy-IPLOC and competence, respectively) up to -0.26 (ratio between items 4 and 9 – autonomy-choice; items 18 and 13 – relatedness and autonomy-choice). The factor loadings were statistically significant. The R-squared (**Table 2**) showed values ranging between 0.27 (item 5, autonomy-volition) and 0.89 (item 19, relatedness). **Table 3** presents the correlations between factors or dimensions, where the highest value corresponded to the association between autonomy-volition and autonomy-IPLOC (*r*_xy_ = 0.90) and the lowest coefficient was observed between autonomy-volition and autonomy-choice (*r*_xy_ = 0.59). In the main diagonal of the matrix, the AVE is also offered for each of the factors. In all cases, the value of AVE is above the threshold of 0.50.

**Table 2:** Factor loadings.

Items	R-squared
1	0.659
2	0.454
3	0.833
4	0.568
5	0.270
6	0.667
7	0.417
8	0.693
9	0.690
10	0.829
11	0.835
12	0.792
13	0.882
14	0.839
15	0.782
16	0.854
17	0.799
18	0.818
19	0.892
20	0.868

*Significance* = *0.05.*

**Table 3:** Correlations between dimensions and average variance extracted.

Dimensions	Competence	Aut-choice	Aut-IPLOC	Aut-volition	Relatedness
Competence	0.79				
Aut-choice	0.72	0.75			
Aut-IPLOC	0.84	0.71	0.70		
Aut-volition	0.69	0.59	0.90	0.60	
Relatedness	0.69	0.64	0.73	0.74	0.72

*Aut, autonomy; average variance extracted in diagonal; p* < *0.05 for all correlations*.

The model returned the following fit indices: the ratio between the χ^2^ (499.68) and its degrees of freedom (160) was 3.12; RMSEA was 0.06 (IC 90%; 0.06–0.07); NNFI 0.96; and CFI 0.97.

Finally, evidence of validity of the BNSSS was obtained in relation to another measuring instrument, the PNTS, that measures frustration of the three psychological needs (competence, autonomy, and relatedness) The correlation observed between the competence factors of both measuring instruments was -0.34 (*p* < 0.001); between autonomy-choice, autonomy-IPLOC, autonomy-volition of the BNSSS, and autonomy of the PNTS of -0.28, -0.36, and -0.46, respectively (*p* < 0.01 in all cases); and between the factor relatedness of both scales of -0.42 (*p* < 0.01).

### The Measurement Model’s Invariance With Respect to Sex and Age

Two groups were formed to collect evidence of invariance in terms of sex, one for men (*n* = 205) and the other for women (*n* = 236). The difference between the CFI values for the configuration model and the invariant factor loadings model was less than 0.01, which was taken as strong evidence of the equality or invariance in the factor loadings for the models featuring male and female athletes. We observed similar results for the differences between the configuration and factor correlation models (ΔCFI = < 0.01).

With respect to age-related invariance, the sample was again arranged into two groups, one for minors (*n* = 277) and the other for adults (*n* = 164). Comparisons of Model 1 against Models 2 and 3 presented ΔCFI values < 0.01. This therefore provided evidence of model invariance between the adult and minor groups.

**Table 4** presents the invariance indices with respect to sex and age.

**Table 4:** Model invariance in terms of sex and age.

	Model	χ^2^	*df*	*p*	NNFI	CFI	RMSEA	RMSEA 90% CI	Δχ^2^	Δ*df*	ΔCFI
Sex	(1) Configuration model	710.54	320	<0.01	0.94	0.95	0.08	0.06–0.08	–	–	–
	(2) Invariant loading factors	740.57	335	<0.01	0.94	0.95	0.07	0.06–0.08	30.03	15	<0.01
	(3) Invariant correlation factors	748.91	345	<0.01	0.94	0.95	0.07	0.06–0.08	38.36	25	<0.01
Age	(1) Configuration model	649.89	320	<0.01	0.95	0.96	0.07	0.06–0.07	–	–	–
	(2) Invariant loading factors	691.95	335	<0.01	0.95	0.96	0.07	0.06–0.07	42.06	15	<0.01
	(3) Invariant correlation factors	693.38	345	<0.01	0.95	0.96	0.06	0.06–0.07	43.49	25	<0.01

*χ^*2*^, chi-squared; df, degrees of freedom; p, p-value; NNFI, Bentler-Bonett non-normed fit index; CFI, comparative fit index; RMSEA, root mean square error of approximation; CI, confidence interval; Δ, difference between values*.

### Reliability Analysis

**Table 5** contains the composite reliability results. All values were greater than 0.70. The highest reliability coefficient corresponded to autonomy-choice and relatedness (0.95) and the lowest was for autonomy-volition (0.87).

**Table 5:** Composite reliability indexes.

Dimensions	Values
Competence	0.88
Autonomy-choice	0.95
Autonomy-IPLOC	0.92
Autonomy-volition	0.87
Relatedness	0.95

## Discussion

The aim of this study was to validate a Spanish version of the BNSSS measurement tool for use in team sports. Taking into account this overall objective and the more specific ones stemming from it (factor validation, invariance analysis and composite reliability analysis), the results indicate that the original version was replicated. Other cultural adaptation, it did not make it. do Nascimento (2015) could not maintain a 20-item structure with appropriate psychometric properties in his Portuguese validation. He obtained a reduced 12-item version of the original scale, with three items for the relatedness dimension, four for competence and five for autonomy; autonomy was grouped within a single dimension as the five-dimensional model presented a poor structural fit. In terms of model fit, results of present study show a good overall fit (Schermelleh-Engel et al., 2003; Levy and Varela, 2006) and are akin to those obtain for the original BNSSS (the ratio between χ^2^ and its *df* = 2.13; RMSEA = 0.06; NNFI = 0.96; and CFI = 0.97). In other versions, such as the Portuguese validation (do Nascimento, 2015), values were less satisfactory and they could not maintain the five-dimensional structure (ratio between χ^2^ and its *df* = 3.18; CFI = 0.91; and RMSEA = 0.08).

A nested model analysis with three models was used to evaluate invariance in sex and age variables. In accordance with Cheung and Rensvold’s (2002) criterion, with respect to sex-related measurement invariance, there were no differences between men and women regarding the factor structure (factor loadings, correlations between factors, and factor variances). This result is consistent with the validation of a Portuguese version of the BNSSS questionnaire (do Nascimento, 2015) which also observed sex invariance. In terms of age, the three models also exhibited invariance.

The composite reliability index yielded good values for each dimension as it exceeded the minimum threshold. Compared to the original version, which measured reliability using Cronbach’s alpha coefficient, the Spanish version validated for team sports features higher reliability values in all dimensions, apart from for competence where both (the original and Spanish) versions recorded values of 0.77. In the Portuguese version with a 12-item structure (do Nascimento, 2015), the Cronbach’s alpha coefficients exceeded the 0.70 threshold established by Nunnally (1967).

In summary, the results of this study have led to the development of the Spanish version of the BNSSS for team sports with good psychometric properties, while maintaining the five-dimensional factor structure first proposed by Ng et al. (2011). As such, the version presented here can be used to evaluate basic psychological needs satisfaction in the context of team sports in Spain. This represents an advance in sports psychology across Spain as we have developed a previously unavailable measurement tool. The scale’s main constraint is that it is limited to team sports because they incorporate the majority of athletes who compete while affiliated to national associations (Spanish National Sports Council, 2017). Future studies should focus on determining whether this model produces similar results in individual sports. With regards to the methodology, alternative statement should be considered for item 5, which presented low factor loading. Perhaps the explanation for this is that item 5 is the unique enunciated in reverse sense (it measures in negative sense: In sport, I feel that I am being forced to do things that I don’t want to do). For last, one methodological limitation to take into account is that the sensitivity and specificity of the scale have not been measured.

## Ethics Statement

This study was carried out in accordance with the recommendations of ‘Ethics committee of Catholic University of Murcia’ with written informed consent from all subjects. All subjects gave written informed consent in accordance with the Declaration of Helsinki. The protocol was approved by the ‘Catholic University of Murcia’.

## Author Contributions

CDF and CA drafted the work or revised it critically for important intellectual content, approved the final version to be published, agreed to be accountable for all aspects of the work in ensuring that questions related to the accuracy or integrity of any part of the work are appropriately investigated and resolved, made substantial contributions to the conception or design of the work, or acquired, analysed, or interpreted the data for the work. FP and MV drafted the work or revised it critically for important intellectual content, approved the final version to be published, and agreed to be accountable for all aspects of the work in ensuring that questions related to the accuracy or integrity of any part of the work are appropriately investigated and resolved. FP made substantial contributions to the conception or design of the work or acquired and interpreted the data for the work. MV made substantial contributions to the acquisition of the data for the work.

## Conflict of Interest Statement

The authors declare that the research was conducted in the absence of any commercial or financial relationships that could be construed as a potential conflict of interest.
